# A Color-Tuning Bioluminescent Sensor (AmyLuc) for Real-Time Monitoring of Intracellular pH Dynamics in Cancer Cells

**DOI:** 10.1021/acs.analchem.6c02204

**Published:** 2026-06-07

**Authors:** Vanessa R. Bevilaqua, Angela Punzo, Alessia Silla, Eliana A. R. Duek, Aldo Roda, Vadim R. Viviani, Cristiana Caliceti

**Affiliations:** † Laboratory of Biomaterials, Department of Surgery, Pontifical Catholic University of São Paulo (PUC-SP), Sorocaba, São Paulo 18030-070, Brazil; ‡ Laboratory of Precision Biochemistry in Aging and Related Pathologies, Department of Biomedical and Neuromotor Sciences, University of Bologna, Bologna 40126, Italy; § INBB—Biostructures and Biosystems National Institute, Rome 00185, Italy; ∥ Laboratory of Biochemistry and Technologies of Bioluminescent Systems, Department of Physics, Chemistry and Mathematics, Federal University of São Carlos (UFSCar), Sorocaba, São Paulo 18052-78, Brazil

## Abstract

Cancer cells show increased glucose uptake and lactate secretion due to mitochondrial respiratory dysfunction and hypoxia, leading to extracellular acidification of the tumor microenvironment (TME) and intracellular alkalinization. This metabolic reprogramming promotes malignant phenotypes, including enhanced invasion, metastasis, multidrug resistance, and immune evasion. Therefore, real-time monitoring of intra- and extracellular pH dynamics is essential to understand tumor progression and to evaluate therapeutic strategies. Here, we report the use of a pH-sensitive bioluminescent color-tuning biosensor, derived from the firefly *Amydetes vivianii* luciferase (AmyLuc), to ratiometrically estimate intracellular and extracellular pH changes associated with metabolic alterations consistent with the Warburg effect in human colorectal adenocarcinoma cells (Caco-2). The ratio of bioluminescence emission intensities at 593 nm (pH 6.0) and 548 nm (pH 8.0) was used to establish a calibration curve for accurate pH determination. Analysis of the green/red emission ratios showed that the treatments with the mitochondrial uncoupler carbonyl cyanide-p-trifluoromethoxyphenylhydrazone (FCCP, 50 μM) and the respiratory chain inhibitor antimycin A (50 μM) induced a sustained intracellular acidification (pH ∼6.3), whereas the extracellular environment showed a gradual alkalinization toward near-physiological pH (∼7.1), consistent with buffering effects of the medium. This intracellular acidification is consistent with metabolic alterations and intracellular proton accumulation caused by the transition from mitochondrial respiration to cytoplasmic anaerobic glycolysis. The results highlight the suitability of AmyLuc as a sensitive color-tuning bioluminescent pH biosensor for real-time monitoring of pH dynamics in cancer cells under metabolic stress and therapeutic interventions.

## Graphical abstract

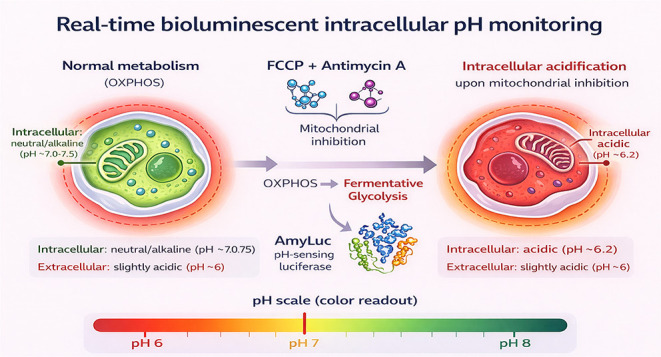

## Introduction

The maintenance of intracellular pH is essential for cellular homeostasis, and its variations often reflect abnormal conditions such as cellular stress or toxic exposure. Changes in intracellular and organelle pH are frequently associated with cellular processes such as division and apoptosis and can reflect pathological states, including inflammation, immune responses, and cancer.^1,2^ Under physiological conditions, the cytosolic and extracellular pH of mammalian cells are similar, typically ranging from 7.2 to 7.4,^3^ whereas organelles such as mitochondria and lysosomes undergo significant pH fluctuations.^4^ Accurate monitoring of pH variations is therefore crucial for tracking both physiological and pathological states.

Many luminescent biosensors and pH indicators have been developed, most of which rely on fluorescence-based intensity measurements,^5–8^ while others employ ratiometric fluorescence strategies to improve measurement accuracy and enable spatiotemporal monitoring of intracellular and extracellular pH dynamics.^9–11^ Although fluorescent sensors are generally affordable and sensitive, they show several disadvantages, including the requirement for external light excitation, which can induce phototoxicity and cause autofluorescence from the biological matrix. Additionally, fluorescent proteins such as green fluorescent protein (GFP) tend to accumulate within cells, potentially interfering with precise, real-time monitoring of intracellular pH changes.^12–14^

The use of bioluminescent pH sensors can circumvent these problems, enabling more accurate and dynamic real-time pH imaging compared to fluorescence-based approaches. For example, bioluminescence resonance energy transfer (BRET)-based sensors named Phlash, which combine a luciferase enzyme with a fluorescent protein, allow sensitive intracellular monitoring without external illumination.^15^ However, BRET sensors require very close proximity between the luciferase–luciferin complex and the fluorescent acceptor, and their interaction can be influenced by factors unrelated to pH, limiting accuracy. A more efficient approach would involve a single luciferase enzyme whose emission spectrum shifts directly in response to pH variations.

We have previously demonstrated that firefly luciferases can serve as bioluminescent color tuning biosensors for intracellular pH and heavy metal concentrations.^16–18^ The luciferase *Macrolampis* firefly has been successfully applied to image pH changes in both the cytoplasm and nucleus.^18^ However, many firefly luciferases, including *Macrolampis* luciferase, are temperature-sensitive; at 37 °C, their emission spectra are already quite red-shifted, reducing the potential spectral resolution. Because firefly luciferases differ in their sensitivity to pH, metals, and temperature, other firefly luciferases have been prospected and their pH-responsive regions have been engineered to optimize these properties.^17,19,20^ Recently we showed that *Amydetes vivianii* firefly luciferase, which displays a stable blue-shifted emission at 37 °C,^21,22^ is a highly suitable reporter gene and pH-sensor for mammalian cells.^23^

Cancer cells predominantly rely on aerobic glycolysis for their energy supply; a metabolic shift associated with impaired mitochondrial respiration and hypoxic conditions. This reprogramming increases glucose uptake and lactate secretion, leading to extracellular acidification (pHe) and a modest intracellular alkalinization (pHi).^24^ Tumor extracellular pH typically ranges from 6.2 to 6.9, in contrast to ∼7.4 in normal tissues,^25–28^ while intracellular pH remains elevated (7.1–7.8). In the case of Caco-2 monolayers under standard conditions, for example, the use of the fluorescent dye BCECF-AM, showed basal intracellular pH values of 7.15 ± 0.03.^29^ This reversed pH gradient is a hallmark of malignancy and promotes proliferation, metabolic adaptation, and resistance to apoptosis.^30^ The acidic tumor microenvironment results primarily from increased lactate production and proton extrusion mediated by monocarboxylate transporters and proton pumps.^31,32^ The acidic extracellular milieu also impairs immune surveillance, facilitating tumor progression. Consequently, therapeutic strategies aimed at normalizing tumor pH have gained increasing attention.^33,34^

Among drugs that affect intracellular pH, FCCP (carbonyl cyanide 4-(trifluoromethoxy)­phenylhydrazone) and antimycin A are known to promote intracellular acidification as part of their effects on mitochondrial function and metabolic stress.^34,35^ FCCP acts as a protonophore, collapsing the mitochondrial proton gradient and disrupting ATP synthesis, while antimycin A inhibits electron transport at complex III, leading to reduced ATP production and increased reactive oxygen species generation.^36^

Given the importance of real-time intracellular pH monitoring during metabolic transitions and therapeutic interventions in cancer, this study aimed to investigate the suitability of the use of a novel color-tuning pH-sensing luciferase from the *A. vivianii* firefly (AmyLuc) to monitor intracellular pH changes in human colorectal adenocarcinoma cells (Caco-2) undergoing metabolic alterations associated with mitochondrial dysfunction and anaerobic glycolytic adaptation following inhibition of the electron transport chain.

## Materials and Methods

### Reagents

Phosphate-buffered saline (PBS) tablets (137 mM NaCl, 2.7 mM KCl, and 10 mM phosphate buffer, pH 7.4), LB broth (Lennox), agar, dimethyl sulfoxide (DMSO), NaCl, KCl, Trizma base, HCl, EDTA, trypsin–EDTA, dithiothreitol, glucose, glycerol, MgSO_4_, ATP, MgCl_2_, CaCl_2_, Na_2_HPO_4_, D-luciferin, antibiotic solution (100×), protease inhibitor cocktail, ampicillin, isopropyl β-d-1-thiogalactopyranoside (IPTG), FCCP, antimycin A, and Hanks’ balanced salt solution were purchased from Sigma-Aldrich (St. Louis, MO, USA). DMEM and phenol red-free DMEM were obtained from Gibco (Grand Island, NY, USA). The Wizard Plus Maxiprep DNA Purification System was obtained from Promega (Madison, WI, USA). *Escherichia coli* XL1-Blue and BL21­(DE3) competent cells were obtained from Agilent Technologies (Santa Clara, CA, USA). Imidazole and nickel–agarose resin were purchased from Qiagen (Hilden, Germany). Human colorectal adenocarcinoma (Caco-2) cells were obtained from ATCC (Manassas, VA, USA). Lipofectamine 3000 was purchased from Invitrogen (Carlsbad, CA, USA).

### Plasmid and Luciferase Gene

The luciferase cDNA from *A. vivianii* was previously codon-optimized for human expression and cloned into the *Hin*dIII site of the pCMV vector to generate pCMV-Amy.^23^ Plasmid DNA was purified using the Wizard Plus Maxiprep DNA Purification System (Promega).

### Luciferase Expression and Purification

Purified luciferase was used for in vitro assay to construct the calibration curve. For such purpose, luciferase was expressed in *E. coli* BL21­(DE3) cells transformed with pCold-Amy, as previously described.^19,21^ Cells were grown in 500 mL LB medium at 37 °C to OD_600_ ≈ 0.4 and induced with 0.4 mM IPTG at 18 °C for 18 h. Cells were harvested (2500*g*, 15 min), and pellets were stored at −80 °C. Pellets were resuspended in extraction buffer (50 mM sodium phosphate, pH 7.0, 300 mM NaCl, 10 mM imidazole) supplemented with protease inhibitor cocktail, lysed by sonication, and centrifuged (12,000*g*, 15 min, 4 °C). The His-tagged luciferase was purified by nickel affinity chromatography and dialyzed against 25 mM Tris-HCl buffer (pH 8.0) containing 10 mM NaCl, 1 mM EDTA, 2 mM DTT, and 10% glycerol.

### Cell Culture

Human colorectal adenocarcinoma cells (Caco-2) were obtained from the American Type Culture Collection (ATCC). Caco-2 cells were grown in DMEM high-glucose medium supplemented with 10% heat-inactivated FBS, 2.5 mM l-glutamine, and penicillin/streptomycin. To perturb mitochondrial function, AmyLuc-transfected Caco-2 cells were treated with 50 μM of the mitochondrial uncoupler FCCP (Sigma C2920) or 50 μM of the mitochondrial complex III inhibitor antimycin A (Sigma-Aldrich A8674). Treatments were always performed in Hanks’ balanced salt solution (HBSS, Sigma H6648, 5 mM glucose). For comparison, additional experiments were also conducted in HBSS supplemented with glucose (25 mM), as described in the Supporting Information.

### Transfection

Caco-2 cells (1 × 10^5^) were seeded in 96-well plates and transfected with pCMV-Amy (containing the humanized firefly luciferase gene of AmyLuc) using Lipofectamine 3000 (Invitrogen, USA) according to the manufacturer’s instructions, generating Caco-2-AmyLuc cells.

### Intracellular and Extracellular pH Evaluation

The day after transfection, the DMEM culture medium was removed and replaced with 100 μL of fresh medium subjected to the indicated treatments. For the intracellular pH analysis, transfected Caco-2 cells were incubated in either DMEM or Hanks’ buffer. Due to the higher buffering capacity of DMEM (25 mM HEPES and 44 mM NaHCO_3_), Hanks’ buffer (4 mM NaHCO_3_) was also employed because its lower buffering capacity allows greater pH responsiveness. Cells were subjected to different treatments: fresh DMEM, DMEM with FCCP or antimycin A, Hanks’ buffer with or without inhibitors, and aged DMEM. After 5 min of treatment and instrument equilibration, bioluminescence measurements were initiated by adding 5 μL of D-luciferin (10 mM) and 5 μL of a solution containing MgSO_4_ (80 mM) and ATP (40 mM), followed by spectral acquisition. For extracellular pH measurements, nontransfected cells were incubated under the same conditions. Prior to the spectral acquisition, 10 μL of purified AmyLuc luciferase (0.36 mg/mL), 5 μL of D-luciferin (10 mM), and 5 μL of a solution containing MgSO_4_ (80 mM) and ATP (40 mM) were added to the extracellular medium, followed by spectral acquisition.

### Bioluminescence Spectra

Bioluminescence spectra were measured using Tecan Spark multiplate plate reader (Männedorf, Switzerland), at 37 °C. The following parameters were chosen to record the spectra: luminescence mode, scanning between 398 and 653 nm, with readings performed in counts/sec, integration time of 1000 ms, and a settle time of 0 ms. Considering that Amy luciferase emits more red light at acidic pH and more green light at alkaline pH (Figure 1A), we first measured BL spectra of AmyLuc in transfected Caco-2 cells (Caco-2-AmyLuc) seeded in media at pH 6.0 and 8.0 and determined the BL spectral peaks (pH 6.0: λ_max_ = 593 nm; pH 8.0: λ_max_ = 548 nm) (Figure 1B). Bioluminescence spectra were acquired from individual wells by sequential spectral scanning under identical acquisition settings throughout the experiments. Repeated measurements were performed over time to monitor pH kinetics while maintaining adequate signal-to-noise ratios.

**Figure 1:**
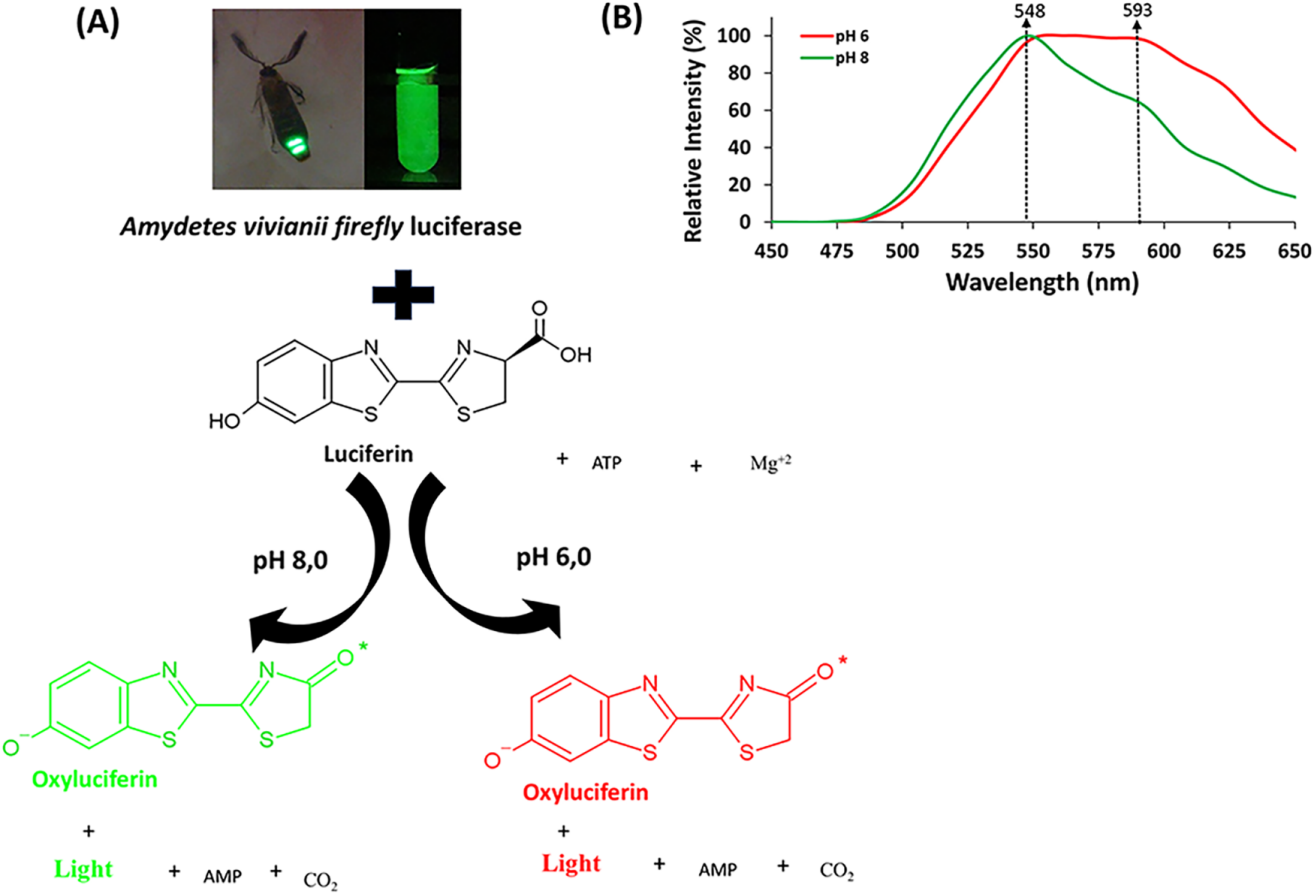
(A) Scheme showing the bioluminescence color of firefly luciferase catalyzed reaction (*A. vivianii*) at pH 6.0 and 8.0. (B) Bioluminescence spectra of AmyLuc transfected Caco-2 cells at pH 6.0 and 8.0, showing the peaks of intensity measured using a Tecan multireader.

### Ratiometric Analysis

To estimate intracellular and extracellular pH, a calibration curve describing the effect of pH on the ratio *I*_548_/*I*_593_ during time was first generated using purified AmyLuc in calibration buffer (135 mM sodium phosphate buffer with 1 mM MgCl_2_, 1 mM CaCl_2_, 10 mM Glucose, 20 mM NaCl at different pHs ranging from 6.5 to 8.0). Briefly, 100 μL of the calibration buffers, 10 μL of purified luciferase (0.36 mg/mL), 5 μL of 10 mM luciferin, 5 μL of 80 mM MgSO_4_ and 40 mM ATP were dispensed in a black 96-well plate, and the bioluminescent reaction was monitored for 105 min (Figure 2 and Table S1). This calibration curve served as a cell-free reference under defined pH conditions. Based on the resulting linear regression between the *I*_548_/*I*_593_ ratio and pH, intracellular and extracellular pH changes were estimated in transfected and nontransfected Caco-2 cells under different treatments. In particular, the *I*_548_/*I*_593_ emission ratio of AmyLuc-transfected Caco-2 cells was used to monitor changes in intracellular *I*_548_/*I*_593_ ratio over time, while extracellular pH changes were simultaneously assessed using purified AmyLuc.

**Figure 2:**
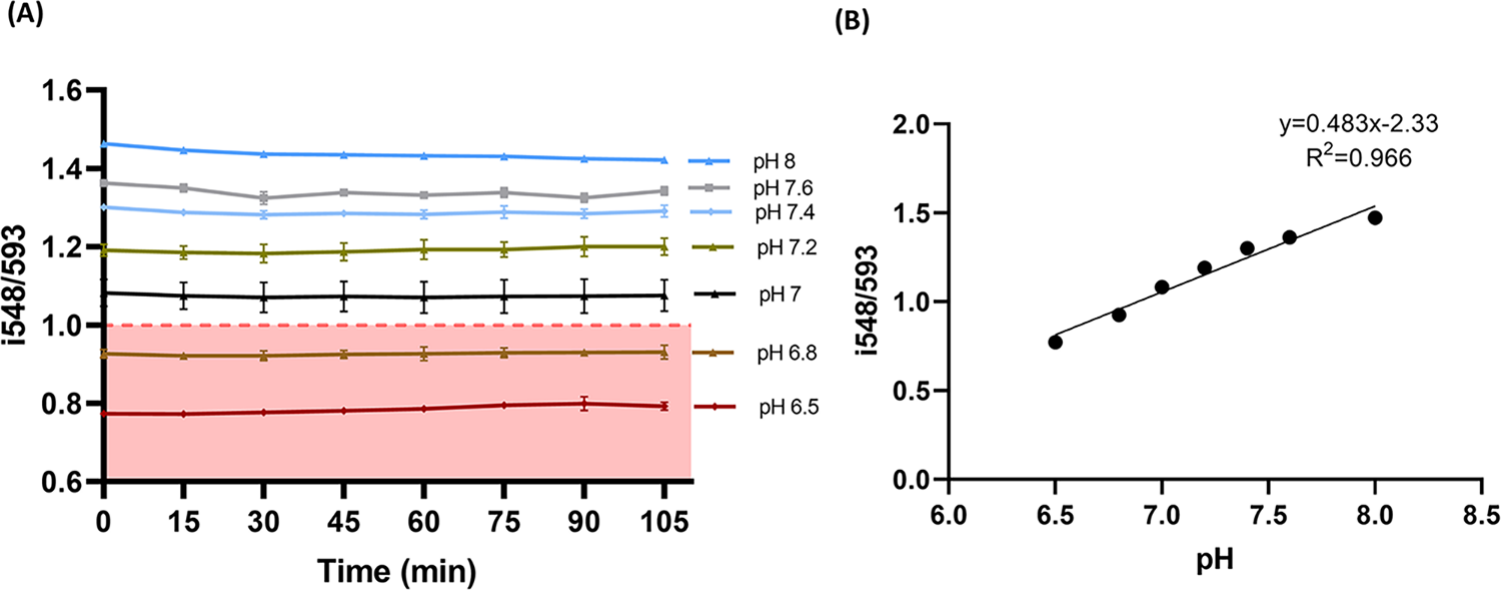
(A) Time course (0–105 min) of the green/red emission ratio (*I*_548_/*I*_593_) at different pH values using purified AmyLuc (cell-free assay). (B) Ratiometric calibration curve obtained at time zero, showing a linear correlation between emission ratio and pH.

### Statistical Analysis

Results are expressed as the mean intensity ratio (*I*_548_/*I*_593_) ± standard deviation of at least three independent experiments. Data were analyzed using GraphPad Prism software, version 8.0.2 (GraphPad Software Inc., La Jolla, CA), and a *p*-value <0.05 was considered statistically significant. Comparisons between two experimental groups were carried out using Student’s *t* test, whereas comparisons among multiple groups were performed using one-way analysis of variance (ANOVA), as appropriate. The limit of detection (LOD) and limit of quantification (LOQ) of the ratiometric pH assay were estimated according to the ICH guidelines^37^ using the equations LOD = 3.3σ/*S* and LOQ = 10σ/*S*, where σ represents the mean standard deviation of three replicate *I*_548_/*I*_593_ measurements obtained across the calibration range (pH 6.5–8.0) at time 0 min, and *S* corresponds to the slope of the calibration curve (0.4746).

## Results and Discussion

### Calibration Curve with AmyLuc

A calibration curve was first constructed using the purified pH-sensitive luciferase from *A. vivianii* firefly (AmyLuc) to enable ratiometric monitoring of pH changes within the 6.5–8.0 range, based on the ratio of green to red emission intensities (*I*_548_/*I*_593_).

Figure 2A shows the time course of the emission ratio of the *in vitro* bioluminescence reaction at different pH values. The ratio remained stable over time (0–105 min) across the tested pH values, suggesting minimal influence of signal decay or reaction kinetics on the ratiometric readout.

A calibration curve was generated by correlating the emission ratio with pH values at time zero (Figure 2B). The operational detection range of the sensor was defined as pH 6.5 and 8.0, beyond which deviations from linearity were observed. According to Figure 2A, Table S1 and to the estimated limits of detection (LOD = 0.10 pH units) and of quantification (LOQ = 0.30 pH units), differences of less than 0.2 pH units, could be estimated using this ratiometric analysis.

Because AmyLuc bioluminescence does not require external excitation light, it avoids photobleaching and other artifacts commonly associated with fluorescence-based pH probes, making it well suited for repeated measurements during long-term kinetic monitoring.

### Evaluation of Basal Intracellular and Extracellular pH in Caco-2 Cells

The basal intracellular and extracellular pH values were evaluated in Caco-2 cells under different buffering conditions. Ratios below 1.0 indicate intracellular acidification, whereas values above 1.0 indicate alkalinization. Although pH is a logarithmic scale, the emission ratio (*I*_548_/*I*_593_) showed an approximately linear response between pH 6.5 and 8.0 (*R* ^2^ ≈ 0.95), enabling calibration and estimation of pH values within this range. Previous studies have reported a linear relationship for this luciferase within the pH range.^23^ pH values obtained in this study by extrapolation outside the pH range 6.5–8.0 should be interpreted cautiously.

When the cells were maintained in DMEM, the intracellular pH remained relatively stable throughout the experiment (0–105 min), ranging from 7.07 to 7.27. These values are consistent with those reported for cancer cells (7.1–7.8).^25–28^ However, the estimated extracellular pH was more alkaline (7.74–7.78), higher than values typically reported in tumor microenvironment studies (6.2–6.9).

In contrast, when Hanks’ buffer was used, cells exhibited an initial intracellular pH of 6.67, followed by stabilization between 6.64 and 6.87 after 30 min (Figure 3), with quantitative values summarized in Table S3. The slightly lower basal intracellular pH observed under Hanks’ buffer conditions, compared to the values typically reported for cancer cells under standard culture conditions,^25–29^ could be associated with the lower bicarbonate buffering capacity and metabolic support of this medium. Under the same conditions, extracellular pH increased from 6.56 to 7.14, remaining closer to the values reported in the literature for these cells.

**Figure 3:**
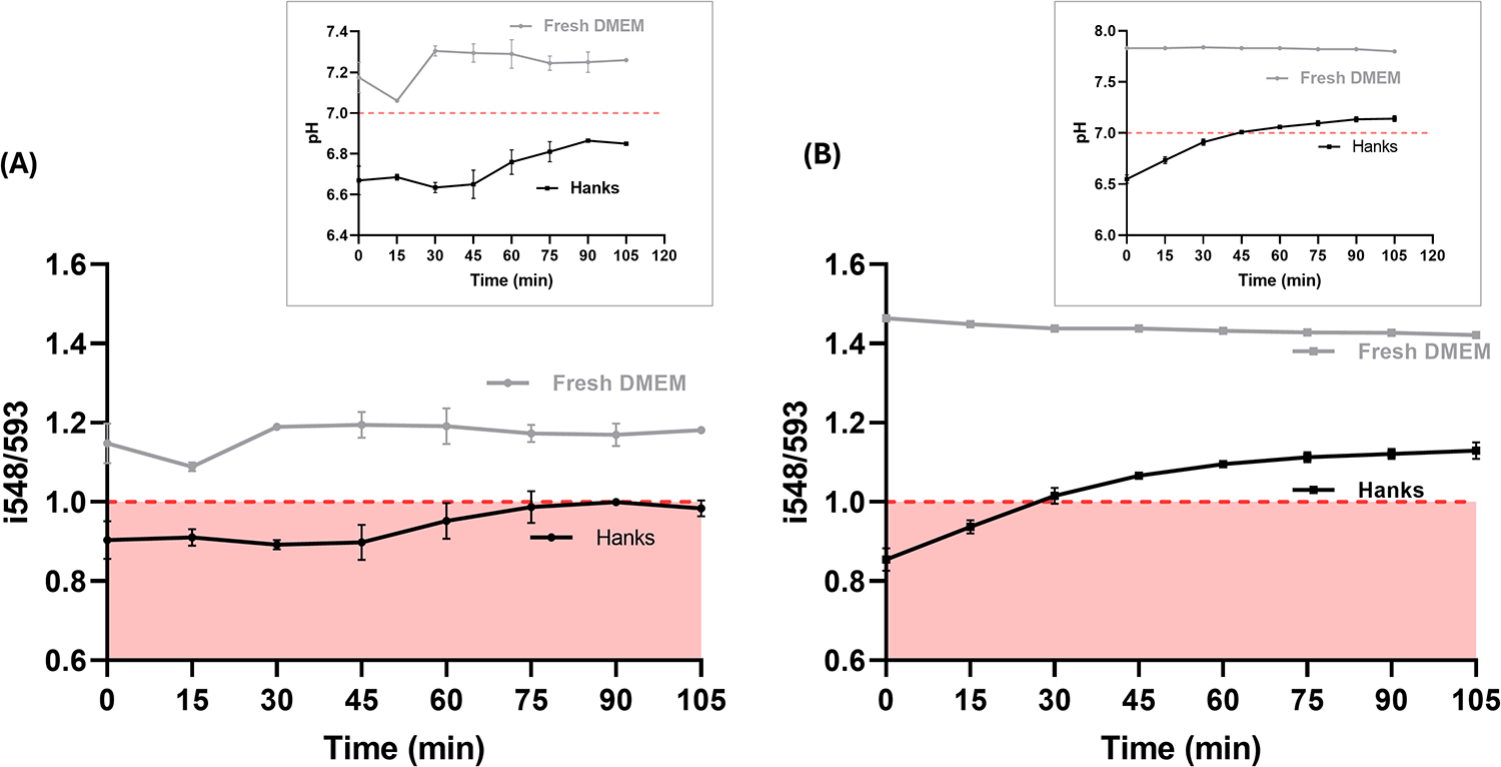
Time-dependent changes in intracellular and extracellular pH and corresponding bioluminescence emission ratios in Caco-2 cells incubated in DMEM or Hanks’ buffer. Upper panels show the estimated (A) intracellular and (B) extracellular pH values over time (0–105 min), calculated from the corresponding green/red emission ratios using the calibration equations derived from Table S2. Lower panels show the corresponding bioluminescence green/red emission ratios (*I*_548_/*I*_593_). Lower *I*_548_/*I*_593_ ratios correspond to more acidic conditions, whereas higher ratios correspond to more alkaline conditions. The shaded red area highlights acidic conditions. Data are presented as mean ± SEM (*n* = 3 independent experiments, each performed with technical replicates).

The differences observed between DMEM and Hanks’ buffer likely reflect the higher buffering capacity of DMEM, which limits the detection of extracellular pH variations, whereas the lower buffering capacity of Hanks’ buffer enhances sensitivity to pH changes. Based on these observations, Hanks’ buffer was selected for subsequent experiments to enhance sensitivity to extracellular pH variations

### Effects of FCCP and Antimycin A in Intracellular and Extracellular pH

The impact of the mitochondrial uncoupler FCCP and the electron transport chain inhibitor antimycin A on intracellular and extracellular pH was assessed during 105 min in Caco-2/AmyLuc cells using the *I*_548_/*I*_593_ ratios derived from the calibration curve (Figure 4 and Table S3).

**Figure 4:**
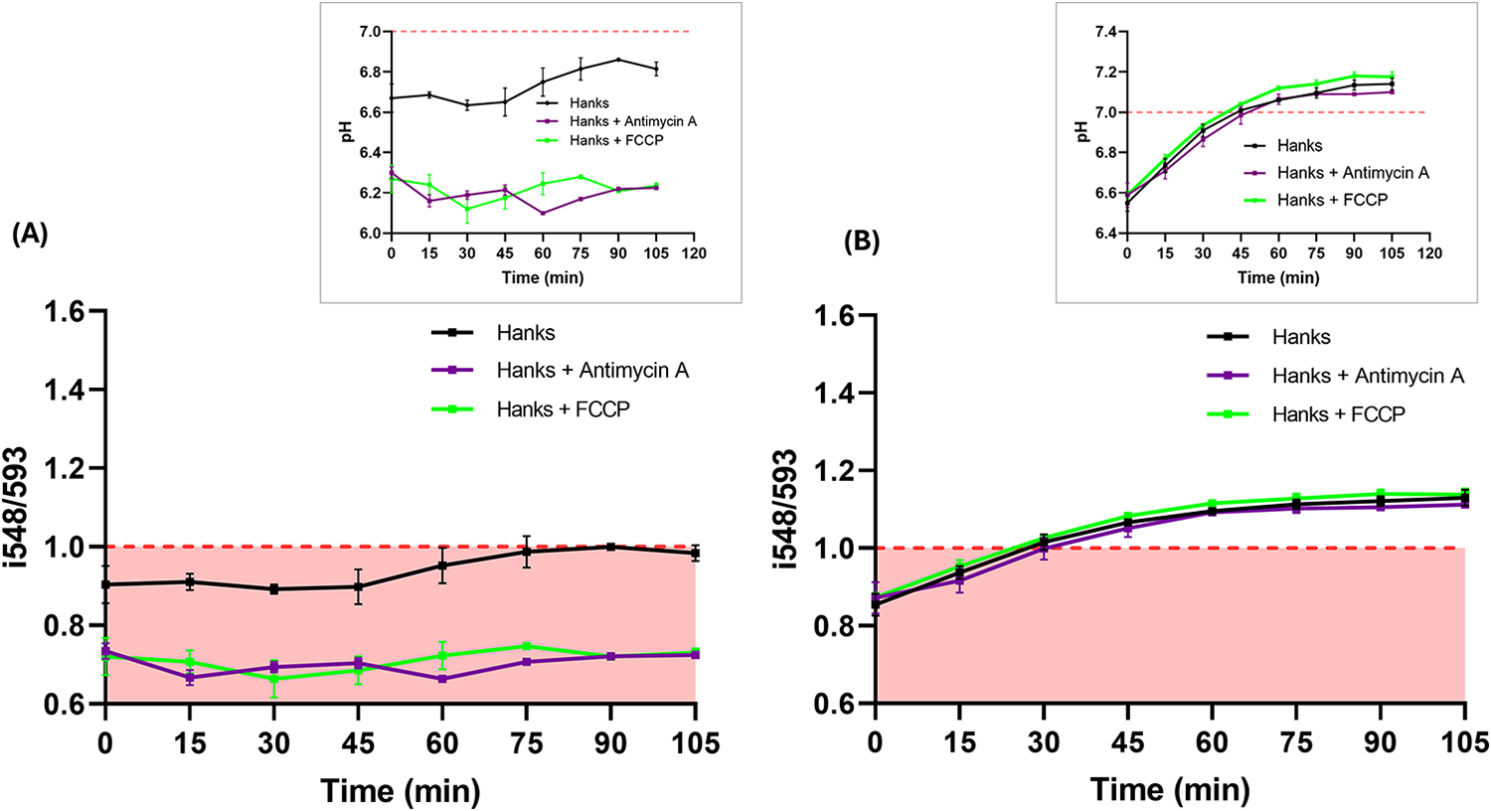
Time-dependent changes in intracellular and extracellular pH and corresponding bioluminescence emission ratios in Caco-2/AmyLuc cells treated with mitochondrial inhibitors in Hanks’ buffer (5 mM glucose). Upper panels show the estimated (A) intracellular and (B) extracellular pH values over time (0–105 min), calculated from the corresponding green/red emission ratios using the calibration equations derived from Table S2. Lower panels show the corresponding bioluminescence green/red emission ratios (*I*_548_/*I*_593_) in cells treated with FCCP or antimycin A. Ratios below 1.0 indicate more acidic conditions, whereas ratios above 1.0 indicate more alkaline conditions. The shaded red area highlights acidic conditions. Data are presented as mean ± SEM (*n* = 3 independent experiments).

#### Intracellular pH

Upon incubation with FCCP (pH 6.28) and antimycin A (pH 6.31), a marked decrease in the emission ratio was observed compared to untreated control cells (pH 6.67), corresponding to intracellular acidification of 0.39 and 0.36 pH units, respectively (Table S3). This response is consistent with the rapid action of these compounds.

At the earliest measured time point (T0), both FCCP and antimycin A had already reduced the intracellular pH, indicating the fast effect of these drugs. This observation is in agreement with previous reports describing rapid dissipation of mitochondrial membrane potential (Δψ_m_) induced by FCCP and fast mitochondrial perturbations caused by antimycin A.^38,39^

After this initial acidification phase, the intracellular pH values tended to stabilize over time rather than showing a continuous decline, suggesting the involvement of cellular pH regulatory processes that may counteract further acidification.

In comparison with the rapid collapse of mitochondrial membrane potential reported in the literature, intracellular pH changes occurred more slowly, reflecting the contribution of intracellular buffering systems and proton extrusion mechanisms operating on longer time scales. The observed intracellular acidification following FCCP and antimycin A treatments is consistent with a metabolic shift from oxidative phosphorylation to anaerobic glycolysis, leading to increased proton and lactate production. According to the literature, the inhibition of the electron transport chain by antimycin A and mitochondrial uncoupling by FCCP lead to intracellular acidification due to increased glycolytic flux and lactate accumulation.^35,40^ This trend was also observed in our experiments using Hanks’ buffer with low glucose concentration (Figure 4), with corresponding values reported in Table S3.

Additionally, FCCP may directly contribute to cytosolic acidification by transporting protons across mitochondrial membranes.^41^

#### Extracellular pH

At the beginning, the extracellular environment is more acidic but, during the first 15 min, the extracellular pH gradually increased reaching the original pH in both treated and control conditions, indicating that the initial alkalinization is not treatment-specific but likely reflects buffering effects of the Hanks solution (Figure 4 and Table S3). In contrast, the intracellular pH stabilized after approximately 30 min (∼6.2–6.3), highlighting distinct regulatory dynamics between intra- and extracellular compartments.

The net extracellular pH profile reflects the balance between proton export and the buffering capacity of the surrounding medium. In buffered systems such as Hanks’ solution, proton extrusion by cells may cause an initial acidification followed by a gradual alkalinization toward equilibrium, as the excess H^+^ is neutralized by the buffer.^40,41^

This behavior is consistent with previous reports showing that mitochondrial inhibition and uncoupling induce cytosolic acidification followed by compensatory proton export mechanisms.^3,31^ However, under the present experimental conditions, the extracellular pH did not decrease but instead gradually increased over time, indicating that the extracellular buffering effects dominate over proton accumulation in the medium.

Overall, the divergence between sustained intracellular acidification and extracellular alkalinization highlights the interplay between intracellular pH regulation and extracellular buffering effects under the present experimental conditions.

Finally, an additional note should be made regarding the effects of glucose concentration on pH. Glucose supplementation enhanced intracellular and, subsequently, extracellular acidification in untreated and antimycin A-treated cells, whereas FCCP had the opposite effect. These observations are further discussed in the Supporting Information (Figure S1 and Table S3).

## Concluding Remarks

In this study, we showed, for the first time, that the pH-sensing firefly luciferase AmyLuc can be effectively used to ratiometrically monitor real-time intracellular pH dynamics in human colorectal adenocarcinoma cells. Using a multimodal plate reader, intracellular pH changes following treatment with the respiratory chain inhibitor antimycin A and the mitochondrial uncoupler FCCP were monitored over time. This approach enables the investigation of cellular responses reflected in pH dynamics under different pharmacological or environmental conditions. In particular, it could be useful for studying compounds that modulate intracellular pH homeostasis in cancer models.

## Supplementary Material



## Notes

Additional calibration data, regression analysis, and extended pH measurements (PDF)
