# Supplementary material for: A Color-Tuning Bioluminescent Sensor (AmyLuc) for Real-Time Monitoring of Intracellular pH Dynamics in Cancer Cells
Source: Anal Chem. 2026 Jun 7;98(24):18188–94. doi: 10.1021/acs.analchem.6c02204 (PMC13295092; doi:10.1021/acs.analchem.6c02204)
Supplement: Supplementary file 1 [file ac6c02204_si_002.pdf]

A Color-Tuning Bioluminescent Sensor (AmyLuc) for Real-Time Monitoring of Intracellular pH Dynamics in Cancer Cells

Vanessa R. Bevilaqua[1,2,5], Angela Punzo[2,3], Alessia Silla[2,3], Eliana A. R. Duek[1], Aldo Roda[3], Vadim R. Viviani[4], Cristiana Caliceti[2,3]*

[1] Laboratory of Biomaterials, Department of Surgery, Pontifical Catholic University of São Paulo (PUC-SP), Sorocaba, SP, 18030-070, Brazil

[2] Laboratory of Precision Biochemistry in Aging and Related Pathologies, Department of Biomedical and Neuromotor Sciences, University of Bologna, Bologna,40126, Italy

[3] INBB – Biostructures and Biosystems National Institute, Rome, 00185, Italy

[4] Laboratory of Biochemistry and Technologies of Bioluminescent Systems, Department of Physics, Chemistry and Mathematics, Federal University of São Carlos (UFSCar), Sorocaba, SP, 18052-78, Brazil

Table S1. Time course of the effect of pH on the I_{548}/I_{593} ratio in a cell-free system.

pH/ Time (min)	0	15	30	45	60	75	90	105
6.5	0.77	0.77	0.77	0.77	0.78	0.79	0.79	0.79
6.8	0.93	0.93	0.93	0.93	0.94	0.94	0.93	0.94
7	1.11	1.10	1.10	1.10	1.10	1.10	1.10	1.10
7.2	1.20	1.20	1.20	1.20	1.21	1.21	1.22	1.22
7.4	1.31	1.29	1.29	1.29	1.29	1.30	1.29	1.30
7.6	1.36	1.36	1.31	1.34	1.33	1.35	1.32	1.35

8	1.46	1.45	1.44	1.43	1.43	1.43	1.43	1.42

[a]The standard deviation varied between 0.03 and 0.26

Table S2. Regression equations and corresponding slopes for pH estimation at different times

Cell-free analysis		
Time (min)	Equation	R^2
0	y= 0.4746x - 2.2598	0.95
15	y=0.4669x - 2.2126	0.95
30	y=0.4482x - 2.0851	0.95
45	y=0.4528x - 2.1129	0.95
60	y=0.4457x - 2.0601	0.95
75	y=0.4433x - 2.0383	0.95
90	y=0.4351x - 1.9849	0.94
105	y=0.4408x - 2.0199	0.94

Table S3. Estimated intra/ extracellular pH values upon different treatments.

Intracellular								
Treatments/Time (min)	0	15	30	45	60	75	90	105
Fresh DMEM	7.14	7.07	7.24	7.26	7.24	7.21	7.27	7.25
Hanks	6.67	6.68	6.64	6.65	6.77	6.84	6.87	6.83
Hanks + Glucose	6.86	6.80	6.75	6.72	6.80	6.78	6.80	6.80
DMEM + Antimycin A	7.18	7.14	7.24	7.23	7.24	7.22	7.19	7.17
Hanks + Antimycin A	6.31	6.17	6.22	6.24	6.16	6.25	6.28	6.29
Hanks + Glucose + Antimycin A	6.32	6.39	6.43	6.31	6.37	6.36	6.33	6.32
DMEM + FCCP	6.99	7.00	7.16	7.20	7.23	7.25	7.28	7.27
Hanks + FCCP	6.28	6.25	6.16	6.21	6.28	6.34	6.28	6.30
Hanks + Glucose + FCCP	7.07	7.11	7.13	7.13	7.20	7.17	7.20	7.21
CRTL-DMEM-aged	6.42	6.45	6.52	6.48	6.48	6.40	6.41	6.43
Extracellular								

Fresh DMEM	7.75	7.74	7.74	7.76	7.77	7.78	7.78	7.78
Hanks	6.56	6.74	6.90	7.00	7.07	7.11	7.12	7.14
Hanks + Glucose	6.40	6.35	6.30	6.23	6.18	6.15	6.14	6.12
DMEM + Antimycin A	7.66	7.67	7.70	7.72	7.75	7.76	7.76	7.75
Hanks + Antimycin A	6.60	6.70	6.87	6.98	7.06	7.08	7.09	7.11
Hanks + Glucose + Antimycin A	6.48	6.40	6.35	6.27	6.19	6.13	6.11	6.14
DMEM + FCCP	7.72	7.72	7.74	7.75	7.77	7.77	7.78	7.78
Hanks + FCCP	6.60	6.77	6.92	7.04	7.11	7.14	7.16	7.16
Hanks + Glucose + FCCP	6.37	6.38	6.43	6.50	6.58	6.64	6.72	6.75
CRTL-DMEM-aged	6.84	6.84	6.83	6.85	6.86	6.87	6.88	6.90
DMEM-Without cells	7.71	7.73	7.77	7.79	7.80	7.80	7.80	7.80

Effect of glucose supplementation on intracellular and extracellular pH

To provide context for the effect of glucose concentration on cellular pH, we compared cells incubated in Hanks' buffer with those maintained in freshly replaced and aged DMEM (Figure 5, main manuscript). The higher glucose content in DMEM was associated with a comparatively more alkaline intracellular pH. Fresh DMEM (25 mM glucose) yielded an initial intracellular pH of 7.14, whereas aged DMEM, in which glucose had been depleted during 3 days of incubation, showed a significantly lower intracellular pH of 6.42 (ΔpH = −0.72).

In contrast, experiments performed in Hanks' buffer, which contains a lower glucose concentration (5 mM), resulted in an intermediate intracellular pH value of 6.67. The greater buffering capacity of DMEM also contributed to maintaining extracellular pH nearly constant at ~7.8, whereas Hanks' buffer showed a progressive increase in extracellular pH over time, reaching values between 6.6 and 7.1 (Table S3).

To further investigate the effect of glucose concentration on cellular pH dynamics, we used Hanks' buffer (5 mM glucose) supplemented with an additional 20 mM glucose to reach a final concentration of 25 mM.

Under these glucose-rich conditions, intracellular pH remained relatively stable at approximately 6.8 during 105 min. Upon treatment with antimycin A, intracellular pH also remained stable but at a more acidic value (~6.32). In contrast, FCCP treatment resulted in a distinct behavior, with intracellular pH slightly increasing from 7.07 to 7.21. These results indicate that, similarly to low-glucose conditions, antimycin A promotes intracellular acidification, whereas FCCP prevents intracellular acidification and stabilizes pH values under glucose-rich conditions (Figure 5, main manuscript; Table S3).

Extracellular pH measurements revealed distinct trends under glucose-rich conditions. In control cells (Hanks' buffer supplemented with 25 mM glucose), the extracellular pH gradually decreased from 6.40 at t_0 to 6.12 after 105 min, indicating progressive acidification. A similar profile was also observed in cells treated with 50 μM antimycin A, where extracellular pH decreased from 6.48 to 6.14 during the same period. In contrast, treatment with 50 μM FCCP led to an increase in extracellular pH from 6.37 to 6.75 over 105 min.

These findings indicate that glucose supplementation sustains intracellular acidification and promotes progressive extracellular acidification in both control and antimycin A-treated cells. In contrast, FCCP treatment promotes extracellular alkalinization and a modest intracellular alkalinization under glucose-rich conditions.

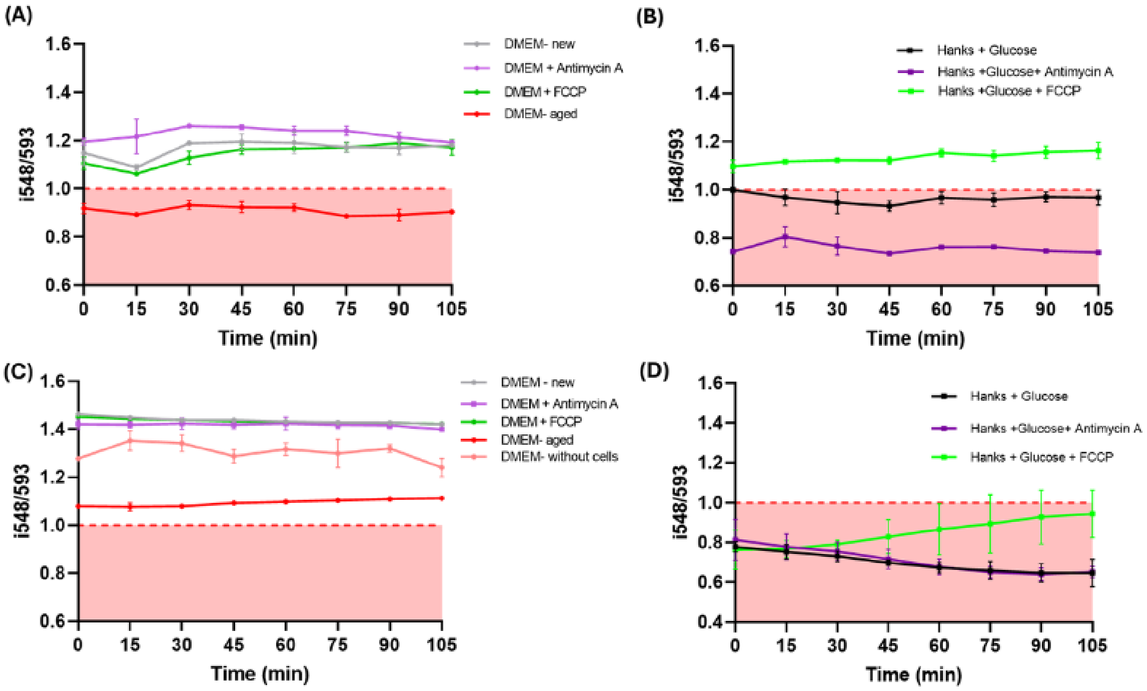

Figure S1. Intracellular (upper panels) and extracellular (lower panels) pH measurements under different media conditions. DMEM was used as control (left panels), while Hanks' buffer supplemented with glucose (25 mM) was used for comparison (right panels).

The acidification of both intracellular and extracellular environments induced by antimycin A under glucose-rich conditions is likely attributable to enhanced glycolysis and lactic fermentation, which increase proton production. Under these conditions, intracellular pH stabilizes around 6.3, while extracellular pH gradually decreases over the course of the experiment (6.48 → 6.14 after 105 min), consistent with continuous proton export.

Considering the larger volume of the extracellular medium relative to the intracellular compartment, even small variations in extracellular pH (e.g., ~0.1 units) may be physiologically relevant. At lower glucose concentrations, extracellular pH gradually increased over time due to buffering capacity of the medium, whereas the amplification

of the glycolytic/fermentative pathway upon glucose supplementation led to progressive extracellular acidification.

These observations are consistent with the distinct mitochondrial and cytoplasmic effects of FCCP and antimycin A reported in the literature. Although FCCP inhibits mitochondrial ATP synthesis, it does not block electron transport, allowing continuous NAD^+ regeneration, favoring aerobic glycolisis.[1,2] and contributing to maintain a relatively stable intracellular pH and extracellular alkalinization rather than acidification.

In addition, FCCP may act as a protonophore due to its relatively low pK_a (~6.1-6.3), facilitating rapid equilibration between intracellular and extracellular pH when proton concentration increases in one compartment. This property may counterbalance its uncoupling activity and contribute to the transient intracellular acidification observed prior to the establishment of steady-state pH under glucose-rich conditions.

In contrast, antimycin A inhibits Complex III of the electron transport chain, limiting NAD^+ regeneration and promoting a shift toward anaerobic glycolysis,[3] leading to sustained lactate production and intracellular acidification (~6.3), followed by progressive extracellular acidification (6.5 → 6.1).

RERERENCES

[1] Luengo, A.; Li, Z.; Gui, D. Y.; Sullivan, L. B.; Zagorulya, M.; Do, B. T.; Ferreira, R.; Naamati, A.; Ali, A.; Lewis, C. A.; et al. Increased Demand for NAD^+ Relative to ATP Drives Aerobic Glycolysis. *Mol. Cell* **2021**, *81*, 691–707. https://doi.org/10.1016/j.molcel.2020.12.012.

[2] Titov, D. V.; Cracan, V.; Goodman, R. P.; Peng, J.; Grabarek, Z.; Mootha, V. K. Complementation of Mitochondrial Electron Transport Chain by Manipulation of the NAD^+/NADH Ratio. *Science* **2016**, *352*, 231–235. https://doi.org/10.1126/science.aad4017.

[3] Pena, A.; Sánchez, N. S.; Gonzalez-Lopez, O.; Calahorra, M. Mechanisms Involved in the Inhibition of Glycolysis by Cyanide and Antimycin A in *Candida albicans* and Its Reversal by Hydrogen Peroxide. *FEMS Yeast Res.* **2015**, *15*, fov083. https://doi.org/10.1093/femsyr/fov083.
